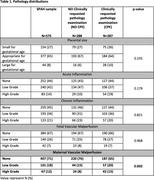# Oral Concurrent Session 2 – Clinical Obstetrics and Quality: Thursday, January 30, 2025 1:30 PM – 4:00 PM

**DOI:** 10.1002/pmf2.12006

**Published:** 2025-01-05

## 19 | Aberrant Fetal Growth in ARRIVE Trial

Bonnie L. Hermann^1^; Cassidy A. O'Sullivan^2^; Fabrizio Zullo^3^; Suneet Chauhan^4^; Hector M. Mendez‐Figueroa^5^; On behalf of the Eunice Kennedy Shriver National Institute of Child Health and Human Development Maternal‐Fetal Medicine Units (MFMU) Network

*^1^UTHealth Houston, Houston, TX; ^2^Christiana Care Health System, Newark, DE; ^3^University of Rome La Sapienza, Rome, Lazio; ^4^Christiana Care, Newark, DE; ^5^McGovern Medical School at UTHealth, Houston, TX*

1:30 PM ‐ 1:45 PM

**Objective**: The primary objective was to compare the rate of cesarean delivery (CD) among individuals with newborns with aberrant growth (i.e. small‐ or large‐for gestational age; SGA or LGA) who were induced versus expectantly managed in the ARRIVE trial. The secondary objectives were to compare the rate of hypertensive disorders of pregnancy (HDP), composite neonatal and maternal adverse outcome (CNAO, CMAO). We hypothesized that the rate of CD would be lower among those with newborns with aberrant growth who were induced versus those expectantly managed.

**Study Design**: A secondary analysis of the ARRIVE trial was performed, with the inclusion criteria of known gestational age and birthweight (BW). Newborns were classified as SGA (BW < 10%) or LGA (BW > 90%), using the nomogram by Alexander et al. CNAO and CMAO are defined in the Table. Relative risks (RR), 95% confidence intervals (CI) and number needed to treat (NNT) were calculated.

**Results**: The rate of aberrant growth was 14.0% (430/3,059) in the induced group and 13.9% (423/3,037) in the expectantly managed group (p = 0.17). CD rate among those with SGA or LGA newborns was significantly lower for those induced (21.6%)  vs those expectantly managed (28.6%; RR 0.75, 95% CI 0.58‐0.99). HDP also differed significantly between the two groups (9.7% vs 16.8%; RR 0.58;  95% CI 0.40‐0.85).The NNT for induction to decrease the rate of CD and HDP among SGA or LGA was 14. The rate of CNAO was similar among the two groups: 7.2% in the induced vs. 7.7% in the expectantly managed group. Likewise, the CMAO—22.6% in the induced group versus 22.3% in the expectantly managed group—did not differ between the groups (p = 0.91; Table).

**Conclusion**: Among low‐risk pregnancies with either SGA or LGA, induction at 39 weeks, compared to expectant management, was associated with 25% reduction in the rate of cesarean delivery and 42% reduction in hypertensive disorder, albeit with no difference in composite neonatal or maternal adverse outcomes. This finding provides support for induction of labor at 39 weeks especially for individuals with suspected neonatal aberrant growth.

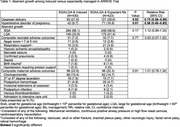

## 20 | Decreased Neuraxial Morphine Dose to Reduce Opioid Side Effects and use After Cesarean Delivery RCT

Ayodeji Sanusi^1^; Yumo Xue^2^; Kevin S. Shrestha^2^; Ayamo Oben^3^; Hanna Hussey^2^; Annalese Neuenschwander^2^; Michelle Tubinis^2^; Mark Powell^2^; Alan T. Tita^2^; Casey Brian^4^

*^1^Center for Women's Reproductive Health, University of Alabama at Birmingham, Birmingham, AL; ^2^University of Alabama at Birmingham, Birmingham, AL; ^3^Maternal Fetal Consultants of Houston, Houston, TX; ^4^West Virginia University, Morgantown, WV*

1:45 PM ‐ 2:00 PM

**Objective**: 1 in 300 opioid naive women become persistent opioid users after cesarean delivery (CD). Regional anesthesia with neuraxial morphine (NM) reduces opioid consumption after surgery. However, >70% of patients have pruritus or urinary retention with the standard 150mcg NM dose. We assessed if reduced NM dose decreases opioid side effects while preserving pain control at CD.

**Study Design**: RCT of 85 patients undergoing scheduled CD at a US tertiary center. Exclusion criteria were quadratus lumborum (QL) block refusal, preeclampsia, insulin‐treated diabetes, placental abnormalities or opioid use disorder. Primary exposure was NM dose. The intervention was 50 mcg NM, bilateral ultrasound‐guided QL block, spinal 12 mg bupivacaine and 15mcg fentanyl. The control group received 150 mcg NM and adjunctive therapies above. Scheduled acetaminophen/NSAIDS and additional opioid therapy for breakthrough pain were available for both groups. Primary outcomes were opioid use 24hours prior to discharge and opioid side effects (recorded medications or urinary retention). Regression models estimated risk ratios/differences for outcomes between groups. Analyses were intent to treat.

**Results**: Of 85 eligible patients, 43(50.6%) were randomized to 50mcg NM and 42(49.4%) to 150mcg NM. The mean age and BMI were 30.3 ± 5.6yrs and 36.0 ± 8.1kg/m^2^, and >95% had a low transverse hysterotomy. There were no significant differences in baseline characteristics between groups. Lower NM dose did not significantly reduce opioid side effects (itching RR 0.55, 95% CI 0.27‐1.10; nausea/vomiting RR 0.90, 95% CI 0.45‐1.80), or use 24 hours prior to discharge (RD ‐1.63, 95% CI ‐8.47‐5.21). Among secondary outcomes, total OMEs were similar between groups. However, intervention group had higher OMEs for parenteral opioids and in the first 24hours, as well as higher recovery room and day of surgery pain scores. No significant differences in other secondary outcomes were noted.

**Conclusion**: Lower NM dose was not associated with significant reductions in opioid side effects or use 24hours prior to discharge.

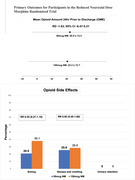

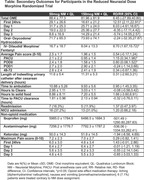

## 21 | Proportion of Time Spent in Category II Fetal Heart Tracing During Labor: Associated Adverse Outcomes

Kristen A. Cagino^1^; Rachel L. Wiley^2^; Aaron W. Roberts^3^; Claudia J. Ibarra^4^; Natalie L. Neff^5^; Kimen S. Balhotra^4^; Khalil M. Chahine^4^; Christina Cortes^4^; Shareen Patel^4^; Tala Ghorayeb^4^; Holly Flores^6^; Fabrizio Zullo^7^; Hector M. Mendez‐Figueroa^4^; Suneet Chauhan^8^

*^1^UT Houston, Houston, TX; ^2^University of California, San Diego, San Diego, CA; ^3^McGovern Medical School at UTHealth Houston, Houston, TX; ^4^McGovern Medical School at UTHealth, Houston, TX; ^5^McGovern Medical School at UT Health, Houston, TX; ^6^University of Texas Health Science Center, Houston, TX; ^7^University of Rome La Sapienza, Rome, Lazio; ^8^Christiana Care, Newark, DE*

2:00 PM ‐ 2:15 PM

**Objective**: To ascertain if the proportion of time spent in Category II fetal heart rate tracing (FHRT) among singleton term (> 37 wks) laboring patients was associated with adverse outcomes.

**Study Design**: Obstetricians—blinded to maternal characteristics and outcomes—reviewed the available FHRT (120 minutes before delivery, at 20 min segments) for all deliveries within a 15‐month period. Term, non‐anomalous, singleton pregnancies who attempted labor were included. We excluded those that only had persistent category I or any segment with category III. Cohort was divided into 3 groups: Category II for < 33% of the time (Group 1), for 33‐66% of the time (Group 2), and > 66% of the time (Group 3). The primary outcome was the rate of composite neonatal adverse outcomes (CNAO); the secondary outcomes were cesarean delivery (CD) for non‐reassuring FHRT and composite maternal adverse outcomes (CMAO). Adjusted odds ratio (OR) with 95% confidence intervals (CI) were calculated.!

**Results**: Among the 5,160 consecutive deliveries, 2,780 (54%) met the inclusion criteria. Of the 321,980 min of FHRT reviewed, 223,000 min (69%) were category II. Specifically, 10% were in Group 1, 26% in Group 2, and 64% in Group 3. Characteristics of FHRT which differed among the 3 groups were frequency of minimal variability, as well as variable, late and prolonged decelerations (Table 1). CD for non‐reassuring FHRT differed significantly between the groups (p < 0.01). The rate of CNAO did not differ among the groups (p = 0.72), however the CMAO differed significantly (p = 0.02). Adjusted OR comparing category II for Group 1 vs. Group 2 and for Group 1 vs. Group 3 indicated that CD for NR‐FHRT, CNAO and CMAO did not differ significantly among the groups (Table 2).

**Conclusion**: Among term deliveries, the majority of FHRTs are category II for over two‐thirds of the total time during the last 120 min of labor. The proportion of time fetal heart rate tracing was in category II did not significantly influence cesarean delivery for non‐reassuring tracing and adverse outcomes for the neonatal‐maternal dyad.

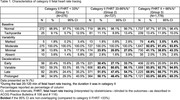

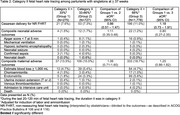

## 22 | Tranexamic Acid Prophylaxis During Cesarean Delivery Among Patients with and Without Hypertension

Adam K. Lewkowitz^1^; Mariam Ayyash^2^; On behalf of the Eunice Kennedy Shriver National Institute of Child Health and Human Development Maternal‐Fetal Medicine Units (MFMU) Network

*^1^Women & Infants Hospital of Rhode Island and Alpert Medical School of Brown University, Providence, RI; ^2^Columbia University Irving Medical Center, New York, NY*

2:15 PM ‐ 2:30 PM

**Objective**: Chronic and pregnancy‐related hypertension (hereafter “HTN”) are associated with increased risk of obstetric hemorrhage and blood transfusion. Should hemorrhage occur among those with HTN, the choice of uterotonics is constrained (methergine is contraindicated). Thus, while prophylactic use of tranexamic acid (TXA) during cesarean delivery (CD) does not alter rates of obstetric hemorrhage or blood transfusion overall, prophylactic TXA during CD may reduce these morbidities among patients with HTN. Our objective was to examine the effect of prophylactic TXA during CD on peripartum bleeding‐related outcomes for patients  by HTN status.

**Study Design**: This is a non‐prespecified secondary analysis of a multicenter, placebo‐controlled trial of patients  undergoing CD who were randomized to 1g of TXA or placebo. For this analysis, all participants were included. The study population was split into subgroups on the basis of HTN prior to delivery. The primary outcome was a composite of maternal death or blood transfusion before hospital discharge or seven days postpartum, whichever occurred first. Blood transfusion was defined as the transfusion of packed red cells or whole blood or cell‐savor autotransfusion device use. Secondary outcomes are in Table 1. Relative risks (RR) were calculated for categorical and mean differences for continuous outcomes. *P* values for interaction with HTN were calculated.

**Results**: Of the 10,995 participants randomized in the parent trial, 2707 (24.6%) had HTN (7.1% chronic hypertension and 17.5% pregnancy related). The primary outcome did not differ among those who received TXA versus placebo in either subgroup (HTN: TXA 4.9% vs placebo 6.0%; RR 0.84 (95% CI 0.62, 1.16); no HTN: TXA 3.2% vs placebo 3.7%; RR 0.91 (0.73, 1.14); *p* for interaction 0.64). For all secondary outcomes, the interaction *p* was non‐significant (all *p* >0.05).

**Conclusion**: The effect of prophylactic TXA during CD on maternal death or blood transfusion within one week postpartum and on other peripartum bleeding‐related outcomes does not differ by the presence of HTN.

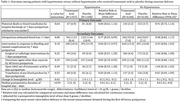

## 23 | Preclinical Development of a Continuous Fetal Lactate Biosensor to Detect Acidosis

Jonathan M. Morris^1^; Arjun Kaushik^2^; Michael Challenor^2^; Lee J. Hubble^2^; Chaitya Shah^2^; Maureen Ross^2^; Ranjusha Rajagopalan^2^; Hassan Mahmood^2^; Allana Gurney^3^; Hayley Powell^3^; Jane Choi^3^; Helen Kershaw^3^; Gabrielle Musk^3^; Jane Pillow^3^

*^1^University of Sydney, New South Wales; ^2^VitalTrace Pty Ltd, Western Australia; ^3^University of Western Australia, Crawley, Western Australia*

2:30 PM ‐ 2:45 PM

**Objective**: Accurate detection of fetal acidosis in labor is an unmet clinical need. Objectives of this study are: 

1. To validate a subcutaneous biosensor for continuous lactate monitoring (CLM) to detect acidosis in a fetal lamb model of asphyxia. 
2. Assess the utility of the CLM biosensor for intrapartum fetal monitoring, by correlating sensor‐detected lactate levels with contemporaneously collected blood lactate values.

**Study Design**: Ewes (n = 3) with singleton pregnancy were anesthetized and the lamb's head was exteriorised. Multiple biosensors were placed onto the lamb's scalp, with electrical current outputs captured. Venous blood samples were collected at regular intervals across the protocol, and lactate and other physiological parameters recorded. After baseline, the umbilical cord was occluded for ten minutes, with recordings continuing over at least three hours during recovery.

**Results**: **Figure 1** shows the relationship between biosensor current (µA), venous blood lactate (mM), and time (s) during the fetal lamb model experiments (animal n = 3; biosensor n = 6), highlighting the utility of the biosensor in monitoring in‐vivo lactate levels. **Figure 1** illustrates a strong correlation between sensor‐measured current and blood lactate levels over time. This is supported by the graphs in **Figure 2 (a‐c)**, with the correlation (R^2^ > 0.88) between sensor current and blood lactate measurements. These results support the capability of the CLM biosensor to detect continuous real‐time changes in fetal lamb lactate concentration, crucial for early detection and management of fetal asphyxia during labor and delivery.

**Conclusion**: This study demonstrates the capability of an innovative subcutaneous biosensor for CLM in detecting early signs of fetal lactic acidosis, highlighted by the strong correlation between sensor‐detected lactate levels and venous blood lactate levels. This technology has the potential to enable continuous fetal lactate measurement, with the ability to revolutionize intrapartum care and improve neonatal outcomes. Ethics and governance approvals are in progress to commence first‐in‐fetus studies.

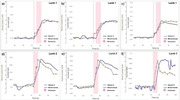

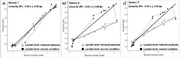

## 24 | After Cesarean Time Interval to Exercise (ACTIVE) Trial: A Randomized Controlled Trial

Brittany J. Roser^1^; James Kinderknecht^2^; Brett Toresdahl^3^; Patricia Ladis^4^; Sonali Iyer^5^; Megan Savage^6^; Timothy Dekker^7^; Paul Christos^5^; Robin B. Kalish^8^

*^1^Stony Brook University Hospital, NY; ^2^Hospital for Special Surgery, New York, NY; ^3^University of Utah Health, Salt Lake City, UT; ^4^WiseBody Physical Therapists, New York, NY; ^5^Weill Cornell Medical College, New York, NY; ^6^Northwell Health at Lenox Hill, New York, NY; ^7^Ascension Medical Group, Rochester, MN; ^8^New York Presbyterian‐ Weill Cornell, New York, NY*

2:45 PM ‐ 3:00 PM

**Objective**: Cesarean delivery (CD) is the most common surgery performed in the United States, accounting for 30% of all births. Currently, there are no clear guidelines on when to initiate exercise after CD. Our objective was to evaluate the impact of early exercise, beginning 2 weeks postpartum, on physical and mental wellbeing.

**Study Design**: We conducted a prospective, parallel‐group, randomized trial (ClinicalTrials.gov NCT04345757) at a single academic center between 2022‐2024. Patients ≥ 18 years undergoing CD were included. Significant maternal or neonatal complications were excluded. Patients were randomized 1:1 to either a video‐based, structured exercise program beginning 2 weeks postpartum or to routine postpartum care, typically avoiding exercise for 6 weeks. The primary outcome was wellbeing at 6 weeks postpartum, assessed using the Patient‐Reported Outcomes Measurement Information System Global Short Form (PROMIS‐GSF).  Secondary outcomes included PROMIS‐GSF physical and mental wellbeing sub‐scores, post‐operative complications and self‐reported breastfeeding (BF) supply. Sample size was calculated to achieve 80% power to detect a 4‐point difference in PROMIS‐GSF score, with α = 0.05. Data was analyzed using two‐sample t‐test and Fischer exact test as appropriate.

**Results**: 846 patients were assessed for eligibility: 646 were excluded, 200 were randomized and 63 completed the primary outcome measure. There was no difference in baseline demographics, obstetric characteristics or baseline PROMIS‐GSF score between groups (Table 1). While there was no difference in 6 week PROMIS‐GSF score, there was a trend towards improved physical health sub‐score in the structured exercise group (Table 2). There were no differences in mental health sub‐score (Table 2), postoperative complication rates or BF supply.

**Conclusion**: Structured exercise two weeks after CD appears to be safe and did not adversely impact physical or mental wellbeing. While low survey completion rate was a limitation, this pilot data will help to inform future research powered to measure the impact of early exercise on enhanced recovery after CD.

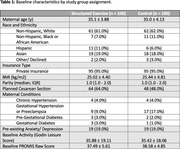

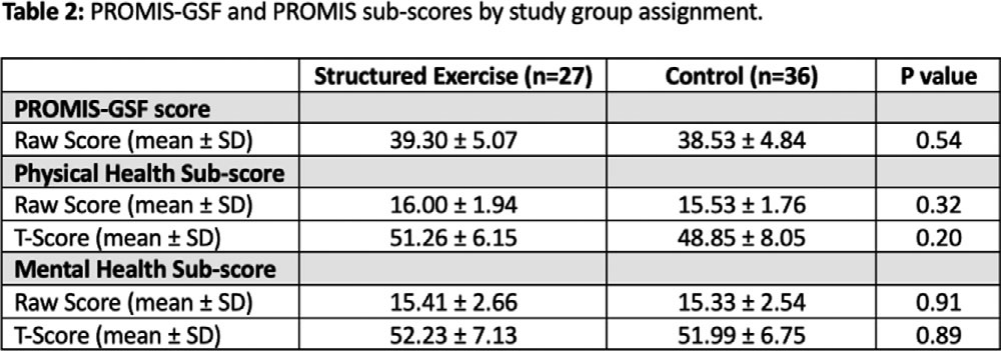

## 25 | Intrauterine Resuscitative Maneuvers for Management of Abnormal Fetal Heart Tracings: A Systematic Review and Meta‐Analysis

Amanda J. Jones^1^; Fabrizio Zullo^2^; Luis Sanchez‐Ramos^3^; Lauren C. Roby^1^; Stephanie Roth^4^; Suneet Chauhan^5^; Matthew K. Hoffman^1^; Anthony C. Sciscione^1^

*^1^Christiana Care Health System, Newark, DE; ^2^University of Rome La Sapienza, Rome, Lazio; ^3^University of Florida College of Medicine, Jacksonville, FL; ^4^Christiana Care Health Services, Newark, DE; ^5^Christiana Care, Newark, DE*

3:00 PM ‐ 3:15 PM

**Objective**: To evaluate the effectiveness of 7 intrauterine resuscitative maneuvers (IRMs)—amnioinfusion, maternal oxygen supplementation, tocolysis, maternal repositioning, discontinuation of labor stimulation, and treatment of maternal hypotension or tachysystole—in mitigating adverse outcomes in the setting of non‐reassuring fetal heart tracings (NRFHT).

**Study Design**: We conducted a systematic review and meta‐analysis of randomized controlled trials (RCTs) of laboring patients with NRFHT randomized to receive one of the 7 IRMs versus no intervention. The primary outcome was Apgar score < 7 at 5 minutes. Secondary outcomes included cesarean delivery rate (CD), CD for NRFHT, relief of NRFHT, umbilical artery (UA) pH < 7.10, UA pH < 7.20, and NICU admission. Summary measures were reported as relative risk (RR) with 95% confidence interval (CI) using the random effects model of DerSimonian and Laird. Higgins I2 > 0% was used to identify heterogeneity.

**Results**: Of 11,372 abstracts reviewed, 8 RCTs were eligible and included: 682 patients randomized to amnioinfusion vs none; 170 randomized to oxygen supplementation vs. none; and 523 randomized to tocolysis before CD vs. none. Likelihood of relief of NRFHT was significantly higher in the amnioinfusion group (68.0% vs 3.27%; RR 18.9; 95% CI 7.16, 50.26) and CD for NRFHT was significantly lower (37.8% vs 54.2%; RR 0.70; 95% CI 0.60, 0.82) vs none. Likelihood of UA pH < 7.20 was significantly lower in the tocolysis group (18.7% vs 35.4%; RR 0.40; 95% CI 0.17‐0.93) vs none. The likelihood of Apgar score < 7 at 5 minutes, UA pH < 7.10, and NICU admission was similar in each group across interventions. We did not identify eligible RCTs for change in maternal position, discontinuation of labor stimulation, or treatment of maternal hypotension or tachysystole.

**Conclusion**: Amnioinfusion for NRFHT was found to relieve NRFHT and reduce CD. Oxygen for NRFHT did not reduce CD or improve fetal outcomes. Tocolysis for NRFHT reduced the rate of rate of fetal acidosis with UA pH < 7.20. No eligible RCTs were found for other IRMs in the setting of NRFHT.

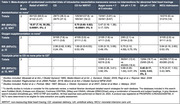

## 26 | Reducing Postpartum Hemorrhage: The Impact of a Standardized High‐Dose Oxytocin Protocol

Evan J. Keil^1^; Elizabeth J. Campbell^2^; Joanne M. Bailey^2^; David E. Arnolds^2^; Diana Peacor^2^; Molly J. Stout^2^; Jourdan E. Triebwasser^1^

*^1^University of Michigan, Ann Arbor, MI; ^2^University of Michigan Medical Center, Ann Arbor, MI*

3:15 PM ‐ 3:30 PM

**Objective**: Oxytocin protocols to prevent postpartum hemorrhage (PPH) vary widely, with limited data suggesting higher doses may reduce PPH. We examined the impact of increasing the dose of oxytocin for active management of the third stage of labor on PPH.

**Study Design**: We performed a single‐center quality improvement project at a large academic medical center. The pre‐intervention phase (PRE) was Jan 1 ‐ Apr 6, 2024 and post‐intervention phase (POST) was Apr 7 ‐ Jul 6, 2024. Quantitative blood loss (QBL) was routinely used throughout. PRE‐oxytocin protocol was 30 units in 500 mL given over 1 h followed by 3.6 units over 1 h. POST‐protocol was 60 units in 1 L given over 1 h. Implementation steps included updating medication orders and infusion pumps and providing staff training. Process measures were uterotonic administration between QBL 500mL and 1L and exposure to >18h of oxytocin during labor, which were monitored using weekly control charts. Healthcare rules were applied to detect special cause variation. Relative risks were calculated to assess the intervention's impact.

**Results**: PPH was less frequent POST (14.6% vs. 10.8%, RR 0.74 [95% CI 0.61‐0.90], **Figure**) despite no change in cesarean birth rate (37.9% vs. 35.3%, p = 0.16) or receipt of any oxytocin during labor (37.0% vs. 38.9%, p = 0.34).  Reduction in PPH was most pronounced among vaginal births (8.7% vs. 5.2%, RR 0.60 [95% CI 0.42‐0.86]). The magnitude of change in PPH was similar for cesarean births though not statistically significant  (24.3% vs. 21.1%, RR 0.87 [95% CI 0.69‐1.10]). Reduction in PPH was not attributable to other process measures as there was no special cause variation in uterotonic administration (mean 64%) or Oxytocin for >18h (mean 18%) during the study period.

**Conclusion**: Implementing an oxytocin protocol of 60 units given over 1 hour reduced overall PPH rates, particularly for vaginal births.

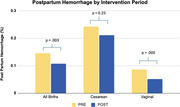

## 27 | Hysterotomy using Barbed vs. Vicryl Suture for Scheduled Cesarean Delivery: A Randomized Controlled Trial

Ayisha B. Buckley^1^; Nicola F. Tavella^2^; Camila Cabrera^2^; Keisha S. Paul^2^; Mariah McKevitt^2^; Monica J. Patel^2^; Lauren A. Ferrara^2^; Jenny Tang^2^; Joanne Stone^3^; Calvin E. Lambert, Jr.^2^; Luciana A. Vieira^4^; Angela T. Bianco^2^

*^1^Weill Cornell Medical Center, New York, NY; ^2^Icahn School of Medicine at Mount Sinai, New York, NY; ^3^Mt. Sinai Medical Center, New York, NY; ^4^Stamford Hospital, Stamford, CT*

3:30 PM ‐ 3:45 PM

**Objective**: Intraoperative reduction of blood loss correlates with reduced morbidity. We examined whether Cesarean Deliveries (CD) had significantly lower quantitative blood loss (QBL) when the hysterotomy was closed with barbed suture.

**Study Design**: This was a single site randomized controlled trial. Patients with a scheduled term CD gave informed consent and were randomly assigned barbed or vicryl suture for hysterotomy closure. Block randomization controlled for primary or repeat CD. We excluded patients with increased risk of hemorrhage (PPH). The primary outcome was QBL (mL). Secondary outcomes were time for hysterotomy closure, additional hemostatic sutures, and postoperative pain (via numerical scoring system). Among covariates for which we adjusted were provider level (Resident, Fellow, Attending) and provider experience with the suture. Multivariable linear regressions estimated adjusted means. A two‐sided ɑ < 0.05 indicated significance. An a priori calculation determined 226 subjects were needed to detect a 30% difference in mean QBL with 85% power.

**Results**: From 2021 to 2023, 226 subjects were assigned barbed (n = 113) or vicryl suture (n = 113). The groups did not differ on demographic or clinical variables. More hysterotomies using barbed suture were done by fellows (*p* = 0.02). The groups differed in provider experience with the suture (*p* < 0.001). Regression models showed a difference in mean QBL between the barbed (521.0 minutes [95%CI: 447.3, 594.7]) and vicryl (558.8 minutes [95%CI: 441.4, 676.2]) groups. There was a shorter time for hysterotomy (4.5 vs 6.1 minutes [95%CI: 4.0, 5.0]) and less postoperative pain (3.3 vs 3.4 [95%CI: 3.0, 3.6]) in the barbed suture group.

**Conclusion**: Closing hysterotomies with barbed suture at scheduled CDs is associated with decreased QBL, postoperative pain, and hysterotomy closure time. These results suggest potential for reduced surgical morbidity and improved patient experience with use of barbed suture. Further studies are needed to corroborate our findings.

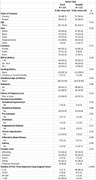

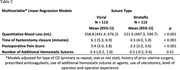

## 28 | Investigating Selection Bias in Research Using Placental Pathology Samples

Linda M. Ernst^1^; Alexa A. Freedman^2^; Renee Odom‐Konja^1^; Lauren Keenan‐Devlin^3^; Greg E. Miller^2^; Ann EB Borders^3^

*^1^Endeavor Health, Evanston, IL; ^2^Northwestern University, Chicago, IL; ^3^Endeavor Health, Evanston Hospital, Evanston, IL*

3:45 PM ‐ 4:00 PM

**Objective**: Increasing research interest has focused on placental pathology and pregnancy outcomes.  Clinically‐requested pathology cases are often used for research due to convenience, which may lead to selection bias. We leveraged a large cohort of prospectively‐collected placentas to compare prevalences of placental pathology in cases with and without a clinically‐requested pathology exam (CPE) and to quantify potential selection bias.

**Study Design**: All placentas were prospectively collected from participants in the Stress, Pregnancy, and Health (SPAH) study, including those with and without CPE. In all cases, placental pathology was categorized and graded in 4 major groups: acute inflammation (AI), chronic inflammation (CI), fetal vascular malperfusion (FVM) and maternal vascular malperfusion (MVM). We compared the distribution of placental pathology in cases with and without CPE. Odds ratios (OR) for preterm birth (PTB) and small for gestational age (SGA) infant were calculated in the whole SPAH cohort and compared with the CPE sub‐cohort. Relative odds ratios (ROR) were used to quantify selection bias. Models were adjusted for sociodemographic and pregnancy characteristics.

**Results**: 575 placentas were collected and examined, 287 with CPE and 288 without CPE. The prevalence of AI, CI and FVM was similar among the 2 groups. However, the prevalence of MVM was significantly higher in CPE placentas, particularly for high grade MVM (15% vs 8%, p< 0.001). In adjusted models, high grade MVM was significantly associated with increased odds of PTB and SGA in the whole SPAH cohort and the CPE sub‐cohort (PTB: SPAH OR = 5.62, CPE OR = 7.56; SGA: SPAH OR = 8.13, CPE OR = 8.25). RORs show a 30% overestimation of the association between MVM and PTB, and only a 2% overestimation in SGA when using a CPE sample.

**Conclusion**: MVM was the only placental pathology seen more frequently in the CPE sample. Research using a CPE only sample may overestimate associations, particularly for MVM and PTB. Because we quantified the selection bias, the RORs from our data can be used to adjust estimates in studies using retrospective CPE samples.